# Human Herpesvirus 8, Southern Siberia

**DOI:** 10.3201/eid1603.091390

**Published:** 2010-03

**Authors:** Olivier Cassar, Sylviane Bassot, Sabine Plancoulaine, Lluis Quintana-Murci, Christine Harmant, Vladimir Gurtsevitch, Natalia B. Senyuta, Larissa S. Yakovleva, Guy de Thé, Antoine Gessain

**Affiliations:** Institut Pasteur, Paris, France (O. Cassar, S. Bassot, S. Plancoulaine, L. Quintana-Murci, C. Harmant, G. de Thé, A. Gessain); Institut National de la Santé et de la Recherche Médicale (INSERM), Paris (S. Plancoulaine); Centre National de la Recherche Scientifique (CNRS), Paris (L. Quintana-Murci); Blokhin Cancer Research Center, Moscow, Russia (V. Gurtsevitch, N.B. Senyuta, L.S. Yakovleva)

**Keywords:** Human herpesvirus 8, HHV-8, epidemiology, molecular epidemiology, genetic variability, Siberia, Russia, viruses, letter

**To the Editor**: Human herpesvirus 8 (HHV-8) is the etiologic agent of Kaposi sarcoma. Sequence analysis of the highly variable open reading frame (ORF)–K1 of HHV-8 has enabled the identification of 5 main molecular subtypes, A–E (*1*). A and C subtypes are prevalent in persons in Europe, Mediterranean countries, northwestern China, and the United States; subtype B, in persons in sub-Saharan Africa; subtype D, in persons in the Pacific Islands and Japan (*2*–*6*); and subtype E, in Native Americans in the United States.

Considering that K1 gene polymorphisms of HHV-8–infected persons reflect the divergence accumulated during the early migrations of modern humans out of Africa (*1*), it is tempting to put the polymorphisms observed in the different subtypes into an evolutionary perspective with their geographic distribution. It is thought that Native Americans infected by subtype E and Pacific Islanders, including those infected by subtype D in the Japanese archipelago, originated from a common ancestral genetic stock in continental Asia. Because Siberia constitutes the geographic link between mainland Asia, North America, and the Pacific (Technical Appendix), it is likely that the Siberian region has served as a source or a corridor of human dispersals to these regions. Thus, we conducted a molecular epidemiology HHV-8 survey of the Buryat population, a major indigenous group in southern Siberia, to gain new insights into the origins, possibly common, of HHV-8 subtypes D and E.

After consent of local authorities and participants, we collected 745 human blood samples in 1995 in 17 medicosocial structures (homes for elderly persons, veterans of the Russian army, hospitalized persons, blood donors) located near Lake Baïkal and originating from Ulan Ude (344), Ust Orda (216), and Chita (185), Siberia, Russia (additional data can be obtained directly from the authors). The median age of those included was 52 years (range 25–98 years); 489 (66%) were women. Antibodies against HHV-8 latency–associated nuclear antigen were identified by immunofluorescent antibody assay by using the BC3 cell line (*3*). Punctuate nuclear staining of BC3 cells at a 1:160 dilution was observed for 187 (25.1%) patients with no difference according to investigated regions (p = 0.32 by χ^2^ test) or between men (25.8%) and women (24.7%) (p = 0.76 by χ^2^ test; Technical Appendix). However, HHV-8 seroprevalence increased with patient age, rising from 12.9% (25–43 years) to 46.4% (>61 years) (p = 1.8 × 10^–13^ by χ^2^ test for trend) (Figure; Technical Appendix). No significant difference was observed in antibody titers according to age (p = 0.45 by Fisher exact test). These results demonstrate that HHV-8 infection is highly prevalent in the Siberian adult population tested.

**Figure:**
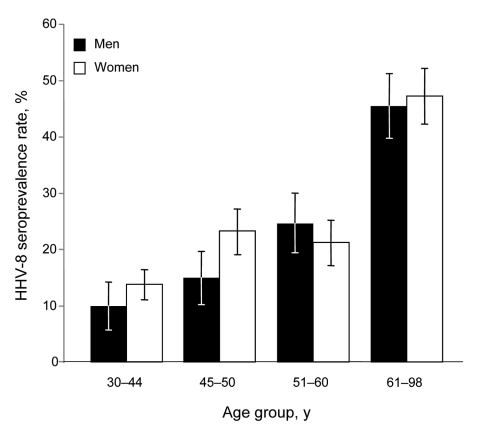
Age-dependent human herpesvirus 8 (HHV-8) seroprevalence rates for 745 persons in southern Siberia 25–98 years of age who lived in the Ust Orda, Ulan Ude, or Chita districts during 1995. Seropositivity was based on strict criteria; only samples showing punctuate nuclear staining clearly reactive at a dilution >1:160 were considered HHV-8 positive. All 187 HHV-8–seropositive samples were tested for antibodies directed against HIV-1/2 by using Genscreen HIV-1/2 Antibody Assay (Bio-Rad Laboratories, Marnes-la-Coquette, France); only 2 were seropositive. Error bars indicate 95% confidence intervals.

HHV-8 infection was determined by nested PCR that amplified a 737-bp fragment of the ORFK1 in peripheral blood buffy coats of 85 HHV-8–seropositive and 10 HHV-8–seronegative persons (*3*). Amplification was positive in 19/85 (22.4%) samples; sequences were obtained for 18 of these samples (Technical Appendix). These sequences showed 0%–7.31% nucleotide divergence and 0%–3.55% amino acid divergence. Nevertheless, 17 strains were found to be closely related with <1.75% nucleotide differences for 684 nt, and only 1 sequence (1445 strain) displayed higher nucleotide divergence.

A comparative sequence analysis, including 66 representatives of K1 gene sequences of the HHV-8 A/C subtypes/subgroups, and sequences obtained from persons originating from Russia, was performed (*7**–**9*). Seventeen of the 18 HHV-8 strains from Siberia belonged to the A subtype; 15 clustered in a newly identified specific subclade (Technical Appendix). Notably, the 1445-Siberian strain, which exhibits the typical 5 aa deletion at positions 201–205, belongs to subtype C and clustered with the 7848 strain previously described by Lacoste et al. (*9*). Furthermore, both strains originate from Chita.

Our results indicate that HHV-8 infection is highly prevalent in the population tested in southern Siberia and extend current knowledge on the worldwide distribution of HHV-8 genotypes. The presence of a Siberian strain monophyletic subclade suggests the existence of HHV-8 strains preferentially spreading among this population in southern Siberia.

To ascertain the maternal ancestry of these persons, we sequenced the hypervariable region I (HVS-I) of the maternally-inherited mitochondrial DNA (mtDNA) and assigned haplogroups on the basis of the HVS-I motifs. Our analyses showed that 17/18 persons analyzed showed a mtDNA motif of clear continental east Asian origin (e.g., A, D correspond to different mtDNA haplogroups). One person (1474-strain) had a lineage (i.e., HV1) that is thought to have a western Eurasian origin. Overall, these mtDNA analyses indicate that the maternal ancestry of the persons examined here can be unambiguously attributed to East Asia, and not to Western Eurasia. K1 subtype A sequences recently found in the Xinjiang Uygur region in China (*10*) do not correspond to the specific Siberian clade described in our study. Thus, we must now consider that the widely distributed HHV-8 A/C subtype, so far mainly observed in Europe and Mediterranean countries, is also largely predominant in continental Asia.

## Supplementary Material

Technical Appendix Map of Siberia showing HHV-8 subtypes, Unrooted phylogenetic tree of human herpesvirus 8 (HHV-8) strains, and Demographic, geographic and serologic data of 19 HHV-8 seropositive persons from Siberia.
