# Supplementary material for: Human Herpesvirus 8, Southern Siberia
Source: Emerg Infect Dis. 2010 Mar;16(3):580–2. doi: 10.3201/eid1603.091390 (PMC3322035; doi:10.3201/eid1603.091390)
Supplement: Technical Appendix — Map of Siberia showing HHV-8 subtypes, Unrooted phylogenetic tree of human herpesvirus 8 (HHV-8) strains, and Demographic, geographic and serologic data of 19 HHV-8 seropositive persons from Siberia. [file 09-1390-Techapp.pdf]

Human Herpesvirus 8, Southern Siberia

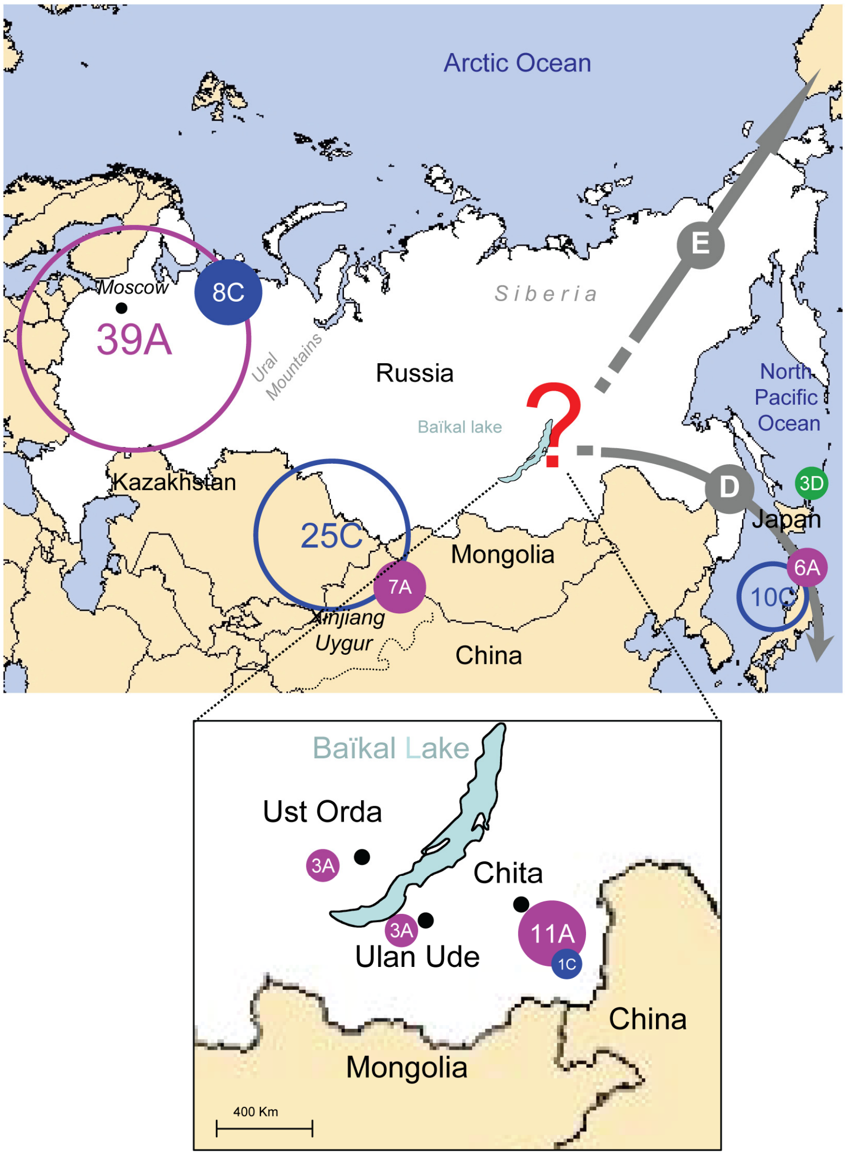

Technical Appendix Figure 1. Map of Siberia (top) showing geographic distribution of human herpesvirus 8 (HHV-8) subtypes according to previous phylogenetic studies based on the complete or partial K1 gene/protein analysis. Russian A/C subtypes strains were obtained from patients with Kaposi sarcoma living in the Moscow area (*1–3*). Chinese A/C strains were obtained in persons originating from Xinjiang Uygur region and reviewed in (*4*). D subtype strains were obtained from inhabitants of Japan, Pacific Islands, and Australia and subtype E strains were found among Native American populations of the Brazilian, Ecuadorian and French Guyanan regions. Gray arrows correspond to the migration routes of human derived from genetic, archaeologic and anthropologic studies. The inset box shows the location of the 3 districts where samples were obtained during this study, Ust-Orda, Ulan-Ude, and Chita, as well as distribution of the molecular subtypes of HHV-8 strains characterized.

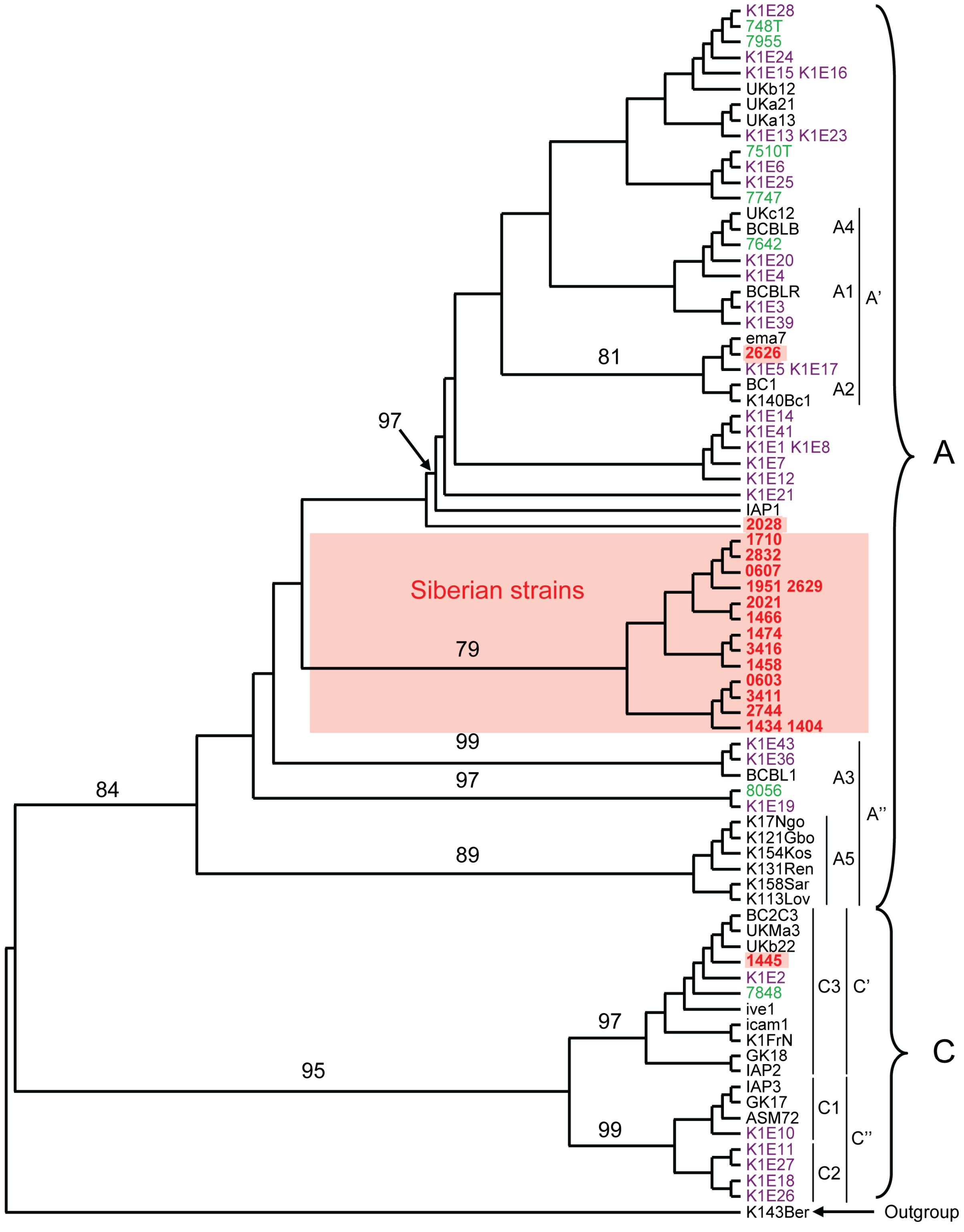

Technical Appendix Figure 2. Unrooted phylogenetic tree of human herpesvirus 8 (HHV-8) strains generated by using the neighbor joining (NJ) method with a 586-bp fragment of the K1 gene. The phylogeny was derived by using the GTR model in PAUP* version 4.0b10 (Sinauer Associates, Inc.,

Sunderland, MA, USA). Reliability of the inferred tree was evaluated by bootstrap analysis on 1,000 replicates. Numbers on each node indicate the percentage of bootstrap samples (1,000) in which the cluster is supported. Only bootstrap values $\geq$75 are given. K143Ber strain was used as an outgroup. The 18 new ORFK1 HHV-8 sequences labeled in red (GenBank accession nos. GQ861475–GQ861492) were analyzed with 66 HHV-8 available sequences from the GenBank database. Previously Russian sequences generated by Lacoste et al. (*3*) and Kadyrova et al. (*2*) are labeled in green and purple, respectively. Bars on the right indicate subtypes, groups and subgroups. A and C correspond to 2 of the 5 main HHV-8 subtypes and A1–A5 and C1--C3 to subgroups within the subtypes described by Zong et al. (*5*). The A′, A″, C′ and C″ grouping reflects the Cook et al. (*6*) classification scheme. Phylogenetic analyses show that all the Siberian sequences, except two pairs (1951/2629 and 1434/1404), are different.

Technical Appendix Table 1. Demographic, geographic and serologic data of 19 HHV-8 seropositive persons from Siberia confirmed by molecular analysis*

Virus strain	Place of origin	Age, y	Sex	Maternal ancestry†	IFA titers (LANA)	PCR K1	HHV-8 molecular subtype	GenBank accession no.
603	Ulan Ude	47	F	East Asia	10 240	+	A	GQ861475
607	Ulan Ude	49	F	East Asia	640	+	A	GQ861476
1710	Ulan Ude	78	F	East Asia	10 240	+	A	GQ861479
1737	Ulan Ude	47	F	East Asia	640	+‡	NA	NA
1404	Chita	66	F	East Asia	2 560	+	A	GQ861487
1434	Chita	64	M	East Asia	320	+	A	GQ861477
1445	Chita	73	F	East Asia	320	+	C	GQ861486
1458	Chita	67	M	East Asia	2 560	+	A	GQ861485
1466	Chita	68	F	East Asia	10 240	+	A	GQ861484
1474	Chita	43	F	West Asia	640	+	A	GQ861478
2626	Chita	83	F	East Asia	10 240	+	A	GQ861482
2629	Chita	44	M	East Asia	640	+	A	GQ861489
2744	Chita	59	M	East Asia	640	+	A	GQ861483
2832	Chita	69	F	East Asia	1 280	+	A	GQ861490
3411	Chita	39	F	East Asia	160	+	A	GQ861491
3416	Chita	35	M	East Asia	160	+	A	GQ861492
1951	Ust Orda	31	F	East Asia	640	+	A	GQ861488
2021	Ust Orda	64	M	East Asia	5 120	+	A	GQ861480
2028	Ust Orda	50	F	East Asia	5 120	+	A	GQ861481

*HHV-8, human herpesvirus 8; IFA, immunofluorescence assay; LANA, HHV-8 specific antibody directed against latent nuclear antigen; PCR K1, amplification of a 737-bp fragment of the ORFK1 genomic region of HHV-8; NA, data not available.
†Genetic feature revealed by mtDNA analysis.
‡Weak PCR signal.

Technical Appendix Table 2. Age-dependent HHV-8 seroprevalence rates, by sex, for 745 persons in southern Siberia 25–98 years of age who lived in the Ust Orda, Ulan Ude, or Chita districts during 1995*

Age group, y	Men n/N (%)	Women n/N (%)	Total n/N (%)
25–43	5/50 (10.0)	23/167 (13.8)	28/217 (12.9)
44–50	9/60 (15.0)	26/112 (23.2)	35/172 (20.3)
51–60	17/69 (24.6)	22/104 (21.2)	39/173 (22.5)
61–98	35/77 (45.5)	50/106 (47.2)	85/183 (46.4)
Total	66/256 (25.8)	121/489 (24.7)	187/745 (25.1)

*HHV-8, human herpesvirus 8. Seropositivity was based on strict criteria; only samples showing punctuate nuclear staining clearly reactive at a dilution ≥1:160 were considered HHV-8 positive.

References

1. Davidovici B, Karakis I, Bourboulia D, Ariad S, Zong J, Benharroch D. et al. Seroepidemiology and molecular epidemiology of Kaposi's sarcoma–associated herpesvirus among Jewish population groups in Israel. J Natl Cancer Inst. 2001;93:194–202. Medline DOI: 10.1093/jnci/93.3.194

2. Kadyrova E, Lacoste V, Duprez R, Pozharissky K, Molochkov V, Huerre M, et al. Molecular epidemiology of Kaposi's sarcoma–associated herpesvirus/human herpesvirus 8 strains from Russian patients with classic, posttransplant, and AIDS-associated Kaposi's sarcoma. J Med Virol. 2003;71:548–56. Medline DOI: 10.1002/jmv.10530

3. Lacoste V, Kadyrova E, Chistiakova I, Gurtsevitch V, Judde JG, Gessain A. Molecular characterization of Kaposi's sarcoma–associated herpesvirus/human herpesvirus-8 strains from Russia. J Gen Virol. 2000;81:1217–22. Medline

4. Fu B, Sun F, Li B, Yang L, Zeng Y, Sun X, et al. Seroprevalence of Kaposi's sarcoma–associated herpesvirus and risk factors in Xinjiang, China. J Med Virol. 2009;81:1422–31. Medline DOI: 10.1002/jmv.21550

5. Zong JC, Ciufo DM, Alcendor DJ, Wan X, Nicholas J, Browning PJ, et al. High-level variability in the ORF-K1 membrane protein gene at the left end of the Kaposi's sarcoma–associated herpesvirus genome defines four major virus subtypes and multiple variants or clades in different human populations. J Virol. 1999;73:4156–70. Medline

6. Cook PM, Whitby D, Calabro ML, Luppi M, Kakoola DN, Hjalgrim H, et al. Variability and evolution of Kaposi's sarcoma–associated herpesvirus in Europe and Africa. International Collaborative Group. AIDS. 1999;13:1165–76. Medline DOI: 10.1097/00002030-199907090-00004
